# Honeybee-Specific Lactic Acid Bacterium Supplements Have No Effect on American Foulbrood-Infected Honeybee Colonies

**DOI:** 10.1128/AEM.00606-19

**Published:** 2019-06-17

**Authors:** Jörg G. Stephan, Sepideh Lamei, Jeffery S. Pettis, Kristian Riesbeck, Joachim R. de Miranda, Eva Forsgren

**Affiliations:** a Department of Ecology, Swedish University of Agricultural Sciences, Uppsala, Sweden; b Clinical Microbiology, Department of Translational Medicine, Faculty of Medicine, Lund University, Malmö, Sweden; c USDA ARS, Beltsville Agricultural Research Center-East, Beltsville, Maryland, USA; University of Manchester

**Keywords:** American foulbrood, *Apis mellifera*, *Bifidobacterium*, *Lactobacillus*, *Paenibacillus larvae*, enzootic disease, honeybee-specific lactic acid bacteria, honeybees, host-pathogen dynamics, tylosin

## Abstract

Paenibacillus larvae, the causative agent of American foulbrood (AFB), is the primary bacterial pathogen affecting honeybees and beekeeping. The main methods for controlling AFB are incineration of diseased colonies or prophylactic antibiotic treatment (e.g., with tylosin), neither of which is fully satisfactory. The search for superior means for controlling AFB has led to an increased interest in the natural relationships between the honeybee-pathogenic and mutualistic microorganisms and, in particular, the antagonistic effects of honeybee-specific lactic acid bacteria (hbs-LAB) against P. larvae. These effects have been demonstrated only on individual larvae in controlled laboratory bioassays. Here we investigated whether supplemental administration of hbs-LAB had a similar beneficial effect on P. larvae infection at colony level. We compared experimentally AFB-infected colonies treated with hbs-LAB supplements to untreated and tylosin-treated colonies and recorded AFB symptoms, bacterial spore levels, and two measures of colony health. To account for the complexity of a bee colony, we focused on (Bayesian) probabilities and magnitudes of effect sizes. Tylosin reduced AFB disease symptoms but also had a negative effect on colony strength. The tylosin treatment did not, however, affect P. larvae spore levels and might therefore “mask” the potential for disease. hbs-LAB tended to reduce brood size in the short term but was unlikely to affect AFB symptoms or spores. These results do not contradict demonstrated antagonistic effects of hbs-LAB against P. larvae at the individual bee level but rather suggest that supplementary administration of hbs-LAB may not be the most effective way to harness these beneficial effects at the colony level.

**IMPORTANCE** The previously demonstrated antagonistic effects of honeybee-derived bacterial microbiota on the infectivity and pathogenicity of P. larvae in laboratory bioassays have identified a possible new approach to AFB control. However, honeybee colonies are complex superorganisms where social immune defenses play a major role in resistance against disease at the colony level. Few studies have investigated the effect of beneficial microorganisms on bee diseases at the colony level. Effects observed at the individual bee level do not necessarily translate into similar effects at the colony level. This study partially fills this gap by showing that, unlike at the individual level, hbs-LAB supplements did not affect AFB symptoms at the colony level. The inference is that the mechanisms regulating the honeybee microbial dynamics within a colony are too strong to manipulate positively through supplemental feeding of live hbs-LAB and that new potential remedies identified through laboratory research have to be tested thoroughly *in situ*, in colonies.

## Abstract (precis)

The previously demonstrated antagonistic effects of honeybee-derived bacterial microbiota on the infectivity and pathogenicity of P. larvae in laboratory bioassays have identified a possible new approach to AFB control. However, honeybee colonies are complex superorganisms where social immune defenses play a major role in resistance against disease at the colony level. Few studies have investigated the effect of beneficial microorganisms on bee diseases at the colony level. Effects observed at the individual bee level do not necessarily translate into similar effects at the colony level. This study partially fills this gap by showing that, unlike at the individual level, hbs-LAB supplements did not affect AFB symptoms at the colony level. The inference is that the mechanisms regulating the honeybee microbial dynamics within a colony are too strong to manipulate positively through supplemental feeding of live hbs-LAB and that new potential remedies identified through laboratory research have to be tested thoroughly *in situ*, in colonies.

## INTRODUCTION

European honeybees, Apis mellifera, are social insects with a diverse microbiota. Recent surveys have shown the presence of dozens of bacterial taxa in adult honeybees, ranging from Gram-positive bacteria to alpha-, beta-, and gammaproteobacteria. While the majority of these bacterial species are not associated with any honeybee disease (1–3), a minority are clearly pathogenic. One of the major pathological threats to honeybees is the spore-forming bacterium Paenibacillus larvae, the causative agent of American foulbrood (AFB), the most lethal brood disease of bees (4–7). Young larvae ingest P. larvae spores with the food provided by the nurse bees. The spores germinate and proliferate in the midgut and invade the larval tissue, where they continue to multiply and produce billions of spores. The spores are extremely resilient and can remain viable for decades (64). The larval remains (scales) are difficult to remove by honeybees and provide a continuous source of infection for new cycles of brood, which, together with the hardiness and viability of the spores, are the key factors driving the lethality and epidemiology of AFB (4, 8). The spores are distributed within colonies by young honeybees performing in-hive tasks, such as cleaning and nursing larvae, and between colonies by swarming, robbing, and in particular by beekeepers moving contaminated material between colonies (9, 10).

There are two main approaches for the control of AFB. The first is to target the long-lived spores by burning the contaminated frames, the hive bodies, and the bees, although the latter can sometimes be saved as an artificial swarm housed on new material (11, 65). The second therapeutic approach is to target the germinating bacteria with antibiotics (e.g., oxytetracycline or the macrolide tylosin), whose prophylactic use in certain countries has inevitably led to antibiotic resistance in P. larvae (12, 13). Such antibiotics mitigate AFB symptoms, thus avoiding regulatory requirements for incineration, but do not kill the bacterial spores, which remain viable in the hive environment. The artificial suppression of clinical symptoms, coupled with a lack of preventative management practices, masks the accumulated potential for disease outbreak (14, 15) without eliminating the causative disease agent (16, 17). The infection pressure from the persistent, infective spores will aid the infection of other colonies and facilitate selection for resistant P. larvae strains.

The recent discovery of the strong antagonistic effect of bacterial microbiota on the infectivity and pathogenicity of P. larvae has identified a new potential approach to AFB control, through the honeybee microbial defenses (18–21). Among the beneficial bacteria associated with honeybees, species belonging to the lactobacilli and bifidobacteria are thought to have health-promoting effects on honeybees, through activating the honeybees’ immune defenses (21, 22), producing antimicrobial compounds inhibiting bacterial competitors (23–25), and efficiently outcompeting pathogenic bacteria for common resources (3, 18, 19). Previously, 13 species of honeybee-specific lactic acid bacteria (hbs-LAB) were isolated from the honey crop of honeybees (26, 27). Both bifidobacteria and lactobacilli are included in the LAB definition due to their similar origin, their organic acid production, and their functional similarity in use by the food and biotech industries (28). hbs-LAB produce a range of metabolites, such as organic acids (27) and extracellular proteins that are secreted upon exposure to various microbial stressors, including lipopolysaccharide and lipoteichoic acid (29). It has been shown repeatedly that hbs-LAB, both individually (23, 25) and in combination (30), have inhibitory effects on P. larvae under laboratory conditions in both microbial inhibition assays and infection bioassays.

However, the honeybee colony is a complex superorganism with both individual and social immune defenses that work in tandem to manage overall colony health but lead to different consequences for infected individuals. Beneficial health effects observed at the individual bee level therefore do not necessarily translate into similar effects at the colony level (31–34). There have been several attempts to assess the effect of beneficial microbes on honeybee health at the colony level (18, 19, 35). Some studies show that beneficial microbes (Lactobacillus rhamnosus) increase honeybee mortality (35), whereas other investigations suggest that beneficial microbes (Lactobacillus acidophilus and Bifidobacterium lactis) affect honeybee colonies positively, increasing honey production (18, 19). In a previous study we demonstrated through laboratory bioassays the inhibitory effects of oral administration of hbs-LAB on P. larvae infection in honeybee larvae at the individual level (36). We have subsequently shown that hbs-LAB inhibit the growth of P. larvae in larvae through extracellular secreted antimicrobial substances (30).

The aim of this study was to determine whether the inhibitory effects of hbs-LAB observed in individual larval bioassays could be replicated in honeybee colonies through oral administration of hbs-LAB supplements and how effective it was relative to antibiotic treatment in inhibiting disease development. We wanted to evaluate the effects of hbs-LAB supplements relative to no treatment (negative control), placebo supplements, or antibiotic (tylosin) treatment on four key parameters: (i) colony-level P. larvae spore prevalence/amounts, (ii) colony-level AFB symptoms, (iii) colony size, and (iv) amount of brood. These objectives were tested at the most realistic organizational scale of the superorganism, namely, at the colony level, and changes in spore levels, clinical symptoms, and colony health were monitored over an entire season. Lastly, the application scheme for the hbs-LAB followed the recommendation of the producer of this commercial product, and the tylosin application followed the current practice in beekeeping. The objectives were tested in both the short term, for the weeks immediately following treatment application, and the long term, covering the entire bee season. For the analysis we chose a Bayesian analytical framework in order to evaluate both the probability and the magnitude of any effect detected and because this approach is ideally suited to analyzing trends in complex study systems, such as honeybee colonies.

## RESULTS

**FIG 1:**
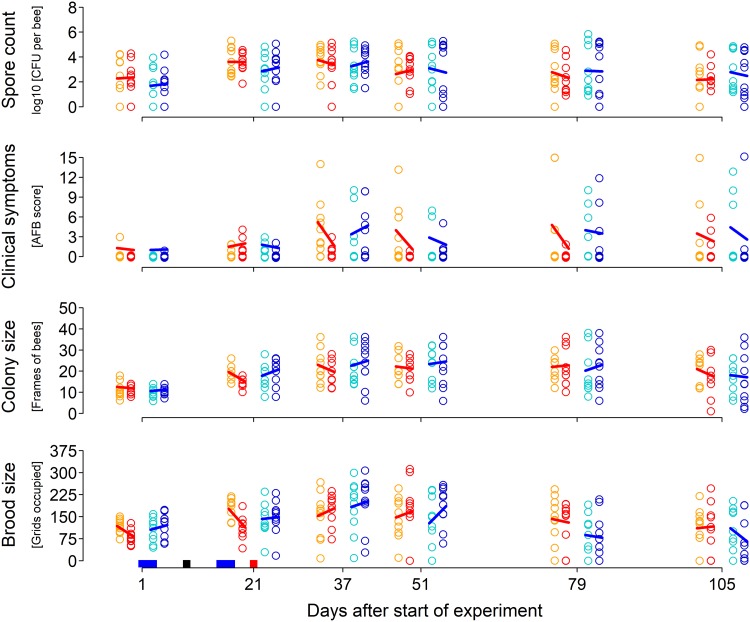
Original data on the effect of tylosin and hbs-LAB on American foulbrood (AFB) and colony strength. Shown are AFB spore counts, clinical symptoms, and colony strength represented by colony size (number of frame sides with bees) and brood size (number of brood) with respect to treatment (control, American foulbrood infection; tylosin, American foulbrood infection and tylosin treatment; placebo, American foulbrood infection and placebo of hbs-LAB; hbs-LAB, American foulbrood infection and hbs-LAB). All six sampling occasions are shown for all 40 colonies, with the lines showing the trends between the mean values for the respective treatment and its control. At the bottom the time course of the treatment application is indicated. Red, tylosin; blue, = placebo/hbs-LAB; black, boost of infection by inoculating all colonies with AFB spores.

The development of the diseases and the colony health are shown in Fig. 1 and reflect possible effect of the treatments as well as natural dynamics of the colony, such as the increasing amounts of brood in the spring and smaller amounts of brood in the autumn. As expected for this complex system, there is great variability among colonies. This is most obvious for the P. larvae spore levels, which differ by several orders of magnitude between different samples.

**FIG 2:**
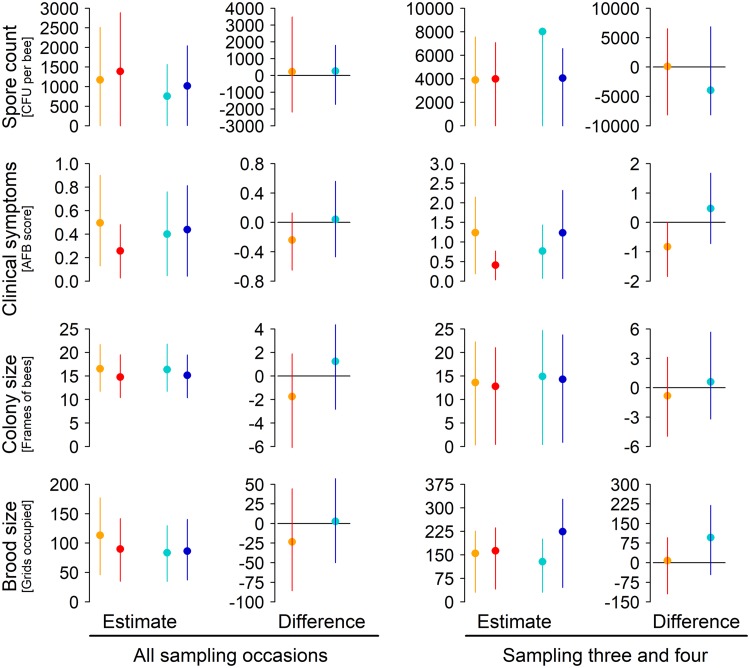
Model predictions of the effect of tylosin and hbs-LAB on American foulbrood and colony strength. Shown are predictions for a whole season (all six sampling occasions) (left side) and predictions for sampling right after the application of the hbs-LAB treatment (right side). The estimates and the difference show the mean value from 1,000 samples of the respective posterior with 89% credibility intervals (highest posterior density intervals). Each difference represents the posterior obtained by subtracting the posterior of one treatment by the posterior of the other treatment.

The output of the Bayesian analyses is shown in Tables 1 and 2 (all pairwise comparisons; see also Fig. S2 in the supplemental material for difference calculation) and Fig. 2 (model estimates and most important pairwise comparison) and consists of a series of predictions (probabilities) of whether a particular parameter would increase or decrease, and by how much, across an entire season (Table 1) or directly after treatment (Table 2).

**TABLE 1:** Pairwise comparison among all treatments in the long term over the whole season (all 6 sampling occasions)^*a*^

Explanatory variable	Combination	%	Mean	Lower CI	Upper CI	*P* [effect > 0]	Effect direction
Spore count	Tylosin–control	18	217	−2,181	3,481	47	↑
	hbs-LAB–placebo	34	260	−1,724	1,799	42	↑
	hbs-LAB–control	−13	−156	−2,184	2,185	53	↓
	Placebo–control	−35	−416	−2,549	1,729	63	↓
	hbs-LAB–tylosin	−27	−373	−2,711	1,724	57	↓
	Tylosin–placebo	84	633	−1,370	3,029	35	↑
Clinical symptoms	Tylosin–control	−48	−0.24	−0.65	0.13	89	↓
	hbs-LAB–placebo	9.5	0.04	−0.47	0.56	41	↑
	hbs-LAB–control	−11	−0.06	−0.57	0.35	64	↓
	Placebo–control	−19	−0.09	−0.52	0.4	72	↓
	hbs-LAB–tylosin	71	0.18	−0.18	0.68	23	↑
	Tylosin–placebo	−36	−0.14	−0.55	0.36	71	↓
Colony size	Tylosin–control	−11	−1.75	−6.08	1.89	79	↓
	hbs-LAB–placebo	8.2	1.24	−2.83	4.36	28	↑
	hbs-LAB–control	−0.9	−0.15	−3.7	3.71	53	↓
	Placebo–control	−8.4	−1.39	−4.62	2.85	73	↓
	hbs-LAB–tylosin	11	1.6	−2.33	4.86	25	↑
	Tylosin–placebo	−2.4	−0.36	−3.6	3.24	57	↓
Brood size	Tylosin–control	−21	−23.51	−85.7	44.47	72	↓
	hbs-LAB–placebo	3.1	2.59	−50.02	57.24	48	↑
	hbs-LAB–control	−24	−27.08	−89.99	37.14	76	↓
	Placebo–control	−26	−29.67	−94.17	30.81	77	↓
	hbs-LAB–tylosin	−4	−3.57	−66.46	51.68	54	↓
	Tylosin–placebo	7.4	6.15	−54.97	55.03	44	↑

a The most relevant comparison, the treatment and its respective control, is shown in the first two rows for each variable. Each comparison represents the posterior density of the differences derived by subtracting the posterior of one treatment from the posterior of the other treatment. The posterior density of the difference is described by the mean and the upper and lower credibility intervals (highest posterior density interval) as well as the difference in percentage and the probability density above zero (*P* [effect > 0]). In the last column the direction of the effect is specified. For example, the first row specifies that there is a 47% chance that the treatment tylosin will lead to an 18% increase in spore counts compared to the control, which is close to the 50% that would occur just by chance.

**TABLE 2:** Comparison among all treatments in the short term for the sampling immediately after the treatment application (occasions 3 and 4)^*a*^

Explanatory variable	Combination	%	Mean	Lower CI	Upper CI	*P* [effect > 0]	Effect direction
Spore count	Tylosin–control	2.3	90	−8,149	6,554	46	↑
	hbs-LAB–placebo	−49	−3,967	−8,156	6,866	53	↓
	hbs-LAB–control	4	156	−7,560	7,402	50	↑
	Placebo–control	110	4,124	−7,519	9,533	47	↑
	hbs-LAB–tylosin	1.7	67	−6,386	8,847	56	↑
	Tylosin–placebo	−50	−4,033	−7,930	7,262	49	↓
Clinical symptoms	Tylosin–control	−67	−0.83	−1.84	0	97	**↓**
	hbs-LAB–placebo	61	0.47	−0.72	1.68	27	↑
	hbs-LAB–control	−0.19	0	−1.33	1.14	56	↓
	Placebo–control	−38	−0.47	−1.38	0.53	83	**↓**
	hbs-LAB–tylosin	200	0.82	−0.27	1.98	7.7	↑
	Tylosin–placebo	−47	−0.36	−1.02	0.45	83	**↓**
Colony size	Tylosin–control	−6	−0.82	−4.95	3.12	68	↓
	hbs-LAB–placebo	4.2	0.6	−3.19	5.69	41	↑
	hbs-LAB–control	9.4	1.28	−2.14	6.23	28	↑
	Placebo–control	5	0.69	−3.47	4.73	38	↑
	hbs-LAB–tylosin	16	2.1	−1.88	7.2	18	↑
	Tylosin–placebo	−11	−1.5	−6.18	1.61	78	**↓**
Brood size	Tylosin–control	5.1	7.86	−119.57	96.41	46	↑
	hbs-LAB–placebo	75	95.91	−45.78	219.67	13	↑
	hbs-LAB–control	45	69.12	−79.44	198.46	21	↑
	Placebo–control	−17	−26.79	−123.35	70.46	63	↓
	hbs-LAB–tylosin	38	61.25	−75.93	191.58	24	↑
	Tylosin–placebo	27	34.66	−66.29	141.04	31	↑

a The most relevant comparison, the treatment and its respective control, is shown in the first two rows for each variable. Each comparison represents the posterior density of the differences derived by subtracting the posterior of one treatment from the posterior of the other treatment. The posterior density of the difference is described by the mean and the upper and lower credibility intervals (highest posterior density interval) as well as the difference in percentage and the probability density above zero (*P* [effect > 0]). In the last column the direction of the effect is specified. For example, in row eight there is a 27% chance that hbs-LAB will increase the symptoms compared to the placebo, meaning that there is a 73% chance that hbs-LAB will decrease the symptoms.

The probability of spore levels going up or down after treatment was about 50% for tylosin, irrespective of whether we looked at the long-term effect over the whole season (Table 1) (47% chance of an increase) or the short-term effect directly after treatment (Table 2) (46% chance of an increase). In contrast, there was a very high (89%) probability of a large (48%) reduction in visible symptoms after tylosin treatment, and this was even higher (97% probability of a 67% reduction) directly after treatment. However, tylosin may come with a moderate long-term cost to colony size (−11%) and brood size (−21%), with 79% and 72% probability, respectively. This detrimental effect was also indicated in the short term for the colony size (68% chance of a decrease), while brood size was not affected immediately (46% chance of an increase).

The trends for the hbs-LAB treatment are slightly different and in many ways more interesting. Like tylosin, the hbs-LAB had no effect on either short- or long-term spore levels. However, hbs-LAB treatment did result in a 27% chance of a 61% short-term increase (meaning a 73% chance of a 61% decrease) in visible clinical symptoms, although this effect dissipated over the long term (41% chance of a 9.5% increase). This (desired) reduction, however, should not be overestimated and cannot be entirely attributed to the hbs-LAB, as the placebo had a similar chance of decreasing the symptoms compared to the control (72% chance of decrease of 19% in the long term and 83% chance of 38% decrease in the short term), and clinical symptoms were similar in hbs-LAB and control colonies (64% chance in the long term and 56% chance in the short term of a difference larger than zero). Only small (4.2%) and not very likely (41% chance) increases in colony size were predicted with hbs-LAB treatment over the short term. However, we detected a tendency of a small decrease in the long term (28% chance of an 8.2% increase, meaning a 72% chance of an 8.2% decrease). The hbs-LAB treatment was also very likely (13% chance of increase, meaning 87% chance of decrease) to decrease the amount of brood by 75% in the short term, but this effect also dissipated over time (48% chance of an increase). In summary, it seemed that the placebo also tended to decrease the clinical symptoms and decreased the colony size and the brood size, but by performing all comparisons, we could show that hbs-LAB is unlikely to have any desired effect but, in contrast, may negatively affect colony health.

## DISCUSSION

Microbial gut symbionts isolated from the honeybee crop are highly specialized in performing metabolic activities necessary to honeybees and could be useful to sustain honeybee health (21, 24, 37). Although several studies have shown favorable effects from hbs-LAB on bee health and their activity against pathogens in individual bees in particular, this does not necessarily correspond to similar effectiveness in the honeybee colony. There are currently very few data available on the effect of supplemental LAB administration on colony-level bee health and performance. The main objective of this study was therefore to test whether the inhibitory effects from hbs-LAB on P. larvae observed in individual bees in laboratory experiments would transfer to honeybee colonies, leading to lower bacterial spore counts and mitigated symptoms of AFB. Additionally, we investigated the effect of hbs-LAB supplements on overall colony strength, i.e., the amount of adult bees and brood. Since AFB is a brood disease, the amount of brood is a particularly relevant colony-level parameter to investigate.

We detected no effect of tylosin or hbs-LAB on the spore levels of P. larvae in the colonies in this study. The results are in line with results from an earlier study by Maggi and coworkers (23), who showed that supplying colonies with organic acids produced by the bacterium Lactobacillus johnsonii CRL1647 did not change the disease dynamics of *Nosema* spp. in honeybee hives despite the inhibitory effects observed in laboratory studies.

It has been demonstrated that antibiotics such as moxifloxacin, ciprofloxacin, linezolid, meropenem, or doxycycline cannot incapacitate Bacillus anthracis (38), which is also a Gram-positive bacterium. As expected, the application of the antibiotic tylosin in our study mitigated visual symptoms of AFB disease, with the most dramatic effect within 2 months of application, but did not decrease P. larvae spore levels either in the short term or over the entire season. The absence of changes in the P. larvae spore levels while visible AFB symptoms decreased clearly illustrates how antibiotic treatment can hide or “mask” the persistent potential for AFB outbreaks. Furthermore, the absence of symptoms increases the likelihood that, through normal beekeeping practices, bees and hive material are exchanged between truly uninfected and asymptomatic spore-containing colonies, thus promoting the “hidden” spread of the disease agent. Prophylactic use of antibiotics for AFB symptom management also facilitates the emergence of antibiotic-resistant strains of P. larvae (9, 13, 39–41). It has been estimated that in areas where antibiotics are used, 10% to 20% of AFB-infected colonies would succumb to the disease if the antibiotic treatment ceased or became ineffective due to the development of antibiotic resistance (42). Beekeeping management techniques to avoid the spread of the disease to other colonies and areas, supplemented by the destruction of symptomatic honeybee colonies, as practiced in many countries, is a more sustainable way to control AFB and to avoid contributing to the development of antibiotic resistance (17, 43).

Another reason for finding better ways for disease control is the harmful effect of the antibiotic on the colony. In our study, we found some indication that tylosin may negatively affect the long-term honeybee colony development, with both colony size and brood size more likely to decrease than increase, relative to the untreated control. These contrasting effects of tylosin (immediate reduction in symptoms versus long-term reduction in colony strength) are difficult to interpret within the context of AFB development. AFB is a devastating brood disease that severely limits the ability of the colony to adequately replace its adult population, leading to dwindling colony size, diminished brood production, and ultimately colony demise. The colony would naturally use all its available homeostatic mechanisms to compensate for this deficit in order to survive, especially in the early stages of the disease, which could include extra emphasis on brood rearing. This may be one indirect explanation for the difference in brood size between treated and untreated colonies. The counterargument, that tylosin directly kills brood at a higher rate than uncontrolled AFB, is more difficult to sustain given the clear observed positive effect on reducing symptoms, which would only benefit brood development and normal population turnover. Surprisingly, the direct effects of antibiotics on colony strength parameters in uninfected colonies are not well documented. One similar study also found that colony size (bee numbers) in AFB-affected colonies decreased by 50% if the colony was treated with tylosin (12), although the responses were quite variable and inconsistent. Although not directly comparable, one study on the application of fumagillin (an antimicrobial agent used to against the pathogen Nosema ceranae [44]) found no difference in bee numbers and brood size between treated and untreated colonies 2 months after application (45). Thus, our study reports the probability and magnitude of negative effects from antibiotic use on colony strength.

The application of hbs-LAB as treatment against AFB does not seem to be a viable option, as neither the spore counts nor the clinical symptoms decreased. Looking only at data from the two sampling occasions directly posttreatment, the probability of finding a decrease in symptoms was higher (a 73% chance) than that of finding an increase (a 27% chance), compared to the placebo treatment. Nevertheless, the hbs-LAB effect did not differ from that of the control, and we interpret this result as being unlikely to change the disease status of a colony. The contrasting results between individual and colony-level applications of beneficial microbes therefore highlight the strength of the honeybee homeostatic mechanisms in neutralizing the effects of any attempt to manipulate the natural conditions of the colony (46, 47). Due to the complexity of a honeybee colony, results obtained in laboratory experiments do not necessarily translate into an effective treatment for honeybee colonies (30).

A recent field study by De Piano et al. (66) showed that honeybee colony performance did not change after administration of Lactobacillus johnsonii AJ5 metabolites. On the other hand, in another field study, administration of a monoculture of Bacillus subtilis subsp. *subtilis* Mori2 to honeybee colonies was shown to stimulate the queen’s egg laying and consequently lead to an increased number of individuals in treated colonies (19). Those data are supported by our study with signs that hbs-LAB application does increase colony strength, since an increase in brood size right after the application was likely (87%) and potentially strong (95.9% [89% credibility interval {CI}, −45.8 to 219.7%] occupied grids with brood, around 75% more than that with the placebo treatment). However, this benefit was short-lived, as over the season it could no longer be detected.

Regarding the effect of the treatments on colony strength, we could show that there is little benefit from applying hbs-LAB, but it is certainly disadvantageous for colony development to apply tylosin. The gut microbiome plays a key role in animal health, including that of honeybees, and the exposure to antibiotics has detrimental effects perturbing the native honeybee gut community (48, 49). The use of the antibiotic tetracycline in honeybee colonies led to elevated mortality of individual adult bees and an increased susceptibility to experimental infection with the opportunistic bacterial pathogen *Serratia* (49). Furthermore, Li and coworkers noticed that perturbation of the gut microbiome by antibiotics weakened the immune function and made honeybees more susceptible to experimental *Nosema* infection (41). Many other studies (3, 23, 50, 51) highlight that the gut microbiome is of great importance for the health of honeybees. This is yet another reason for exploring alternatives to antibiotics for combatting diseases in beekeeping. Most hbs-LAB are highly sensitive to antibiotics such as tylosin and oxytetracycline (52). The long-term use of antibiotics for treatment and control of bacterial diseases in beekeeping perturbs the balance of the honeybee gut microbiome, including hbs-LAB, and affect honeybee health.

The aim of the study was to test hbs-LAB application at the most relevant spatial and temporal scales. However, to gain a mechanistic understanding of the relationship between microbiota and pathogen prevalence in honeybees, future studies will need to better understand the dynamics within a colony. For example recent results indicate that hbs-LAB diversity is very variable over a season, that there is greater diversity in colonies with an AFB history, but that AFB spore levels are unrelated to hbs-LAB levels (S. Lamei et al., unpublished data).

In conclusion, this work does not refute the beneficial nature of honey crop bacteria as has been abundantly shown previously (24, 27, 36, 52), but it does show that translating this knowledge into a useful application is not straightforward and requires careful consideration of the social character and the natural homeostatic mechanisms governing health, microbiome diversity, and function in honeybee colonies (3, 34). Moreover, it confirms that neither supplemental hbs-LAB nor the antibiotic tylosin has short- or long-term effects on the spore levels of P. larvae, the causative agent of AFB, thus maintaining the infection pressure and the risk of disease outbreaks.

## MATERIALS AND METHODS

### Experimental design.

Forty honeybee colonies headed by queens of the same age from a single queen breeder were established on 24 March 2014 using 1.2-kg packages of honeybees in an isolated apiary in Beltsville, MD, USA. The colonies were placed in an apiary with a history of AFB, and the frames and boxes used came from colonies that were managed with AFB in previous studies. All colonies in this apiary would have had some exposure to AFB, but to ensure exposure, all colonies were given a standard spore suspension as described below. The colonies were fed three times with a 1:1 sucrose-water solution to help establish the colonies and promote growth of adult bee populations. The colonies were assessed and inspected on 23 April for the presence of AFB, and AFB severity was rated as noted below. The colonies were assigned to the treatment groups using a stratified random design; i.e., colonies were ranked according to AFB severity and divided into groups of four down the ranking, with subsequent random treatment assignment within each ranking group; each colony in each ranking group received one of the following treatments in a double-blinded fashion: (i) antibiotic (tylosin), (ii) hbs-LAB placebo, (iii) hbs-LAB supplement, and (iv) no treatment (control). All 40 colonies in the AFB apiary were inoculated on 1 May 2014 with P. larvae to boost the onset of AFB, by spraying 2 combs of unsealed brood in each colony with 5 ml of a sucrose solution containing approximately 0.2 × 10^9^ P. larvae spores. The hbs-LAB supplement and the placebo supplement were, as recommended by the producer, administered on two occasions, 23 to 25 April and 7 to 9 May 2014, 1 week before and 1 week after AFB inoculation. The tylosin treatment was administered on 13 May, 12 days after AFB inoculation, as we expected the infection to establish then. The colonies were sampled, assessed, and inspected for AFB symptoms on 6 occasions between April and August 2014: on 23 April (day 1), 13 May (day 21), 29 May (day 37), 12 June (day 51), 10 July (day 79), and 5 August (day 105) (Fig. 1).

### Preparation of the tylosin and hbs-LAB treatments.

SymBeeotic, a proprietary mixture of hbs-LAB species, was used as a supplement in honeybee colonies. This hbs-LAB mixture consisted of 13 viable species of hbs-LAB (Lactobacillus kunkeei Fhon2N, Lactobacillus apinorum Fhon13N, Lactobacillus mellis Hon2N, Lactobacillus mellifer Bin4N, Lactobacillus apis Hma11N, Lactobacillus helsingborgensis Bma5N, Lactobacillus melliventris Hma8N, Lactobacillus kimbladii Hma2N, Lactobacillus kullabergensis Biut2N, Bifidobacterium asteroides Bin2N, Bifidobacterium asteroides Bin7N, Bifidobacterium asteroides Hma3N, and Bifidobacterium coryneforme Bma6N), with a total cell count of 10^9^ CFU/g honey (26, 27, 53), mixed with sterilized (102°C for 30 min) Swedish heather honey. SymBeeotic was prepared and given to the colonies according to the manufacturer’s instructions, as follows. Each SymBeeotic bag was added to 1.5 kg inverted sugar solution diluted with 1 liter of warm water (45°C). This mixture was kept between 30 and 40°C overnight, and around 73 ml of this mixture per colony was used for feeding honeybees. The same preparation and administration procedure was followed for the placebo treatment, which consisted of just sterilized Swedish heather honey, without hbs-LAB. For the antibiotic treatment, 200 mg tylosin tartrate was applied per colony in 20 g of finely powered confectioners’ sugar (Domino sugar 10X).

### Sample collection.

Approximately 200 adult honeybees per sample were collected from the brood chamber in a small cardboard box and stored at −20°C until further analysis.

### Colony assessments.

Colony strength and development were assessed on each of the 6 sampling occasions of the experiment. The amount of brood was assessed by counting the number of 5- by 5-cm sections on both sides of each frame occupied with sealed brood. A single side of one Langstroth frame is equal to 32 such 5- by 5-cm sections (54). The number of adult bees was assessed by counting the number of frame sides covered with adult bees (55). For brood and adults separately, the estimates for each side of all frames were summed to produce composite brood and adult bee scores for the entire colony.

### AFB severity assessment.

The severity of AFB disease was determined by removing each frame and making a visual inspection of the brood (immature larval and pupal honeybees) for evidence of infection. AFB is easy to identify in the field, since the disease symptoms are highly diagnostic. These include partially uncapped cells, a foul odor, and the presence of dead and decaying (pre)pupae within cells. Samples of diseased brood were taken for positive laboratory identification to confirm the field diagnoses. Colonies were rated according to the severity of AFB infection as described by Pettis and Feldlaufer (16); this is the recommended grading system for AFB (56). Each frame side with brood was rated for AFB infection on a scale of 0 to 3: 0, no visible signs of disease; 1, fewer than 10 diseased brood cells; 2, 11 to 100 diseased cells; and 3, more than 100 diseased cells. The scores for each side of all frames were summed to produce a composite AFB score for the entire colony.

### Cultivation of P. larvae from honeybee samples.

Worker honeybee samples were crushed and cultivated on Mueller-Hinton broth–yeast extract–potassium phosphate–glucose–pyruvate (MYPGP) agar plates as described previously (57). The numbers of P. larvae colonies were counted, and the data were presented as CFU per honeybee.

### Data analysis.

The data consisted of 6 sets of measurements with 10 replications (honeybee colonies) for each treatment group, except for 2 colonies that died during the experiment (day 79, placebo; day 105, tylosin). The data were analyzed using multilevel Bayesian linear models, since these models can account for all uncertainty in the data (58, 59). Contrary to the frequentist approach, which calculates the probability of obtaining the observed data given a particular hypothesis (tested with *P* values), the Bayesian approach calculates the probability of the hypothesis given the observed data. The results of the Bayesian models are posterior probability distributions for each of the parameters modeled, from which probabilistic statements about the size and direction of possible effects can be made. Differences between treatments can be calculated by subtracting one posterior distribution from the other (see Fig. S2 in the supplemental material).

All models included the treatment as the main factor to be analyzed. Since in these analyses we were interested primarily in the effect of treatment over the entire season, we included sampling date as a random effect, thereby accounting for the repeated-measure structure of the sampling. We furthermore included colony ID and each observation as random effects (see Appendix for model structures). Differences among the colonies were therefore accounted for and each observation received its own likelihood, meaning model overdispersion was not possible (60). Although the first sampling occasion was prior to both treatment and AFB booster inoculation, we decided to include all 6 sampling occasions in the analyses, since we were interested primarily in the overall effect across the entire season and AFB spores and symptoms were also present on sampling occasion 1, before the AFB booster inoculation, as part of the natural conditions of the AFB apiary (see “Experimental design” above). In order to separate the more immediate and the whole-season effects of the tylosin or hbs-LAB treatments, we build models with different subsets of the data. For these short-term models we included sampling occasions 3 and 4 (days 37 and 51), since these were within 1 month after application of the treatments and effects on the colony are likely to be delayed (49).

In total we built 8 models: 4 with all data from all sampling occasions and 4 with the data from sampling occasions 3 and 4. From the posteriors we calculated the mean value and the 89% credible intervals (highest posterior density intervals; see Fig. S2 for an explanation). We then calculated the differences between the treatments and the control by subtracting the posterior of the control from the posterior of the treatment. In addition, the remaining 4 combinations were also calculated. For each we calculated the absolute difference (mean ± credibility intervals compared to the control values), the relative difference in percentage (with control values as a reference), and the probability that the differences are larger than zero (*P* [effect > 0]; in case of the treatment being smaller than the control, e.g., *P* [effect > 0] = 90% would indicate a 9 in 10 chance of a decrease due to the treatment).

All response variables are counts and therefore received a Poisson likelihood (log link function). Although the AFB score actually represents a mixture of ordinal (0 to 3) and continuous (sum of 0 to 3 per frame side for each colony) scales, here we used the final scores as counts. In all models, we used minimal informative priors (see Appendix) that were updated with the data to arrive at the posterior distributions for each parameter. The Bayesian Markov chain Monte Carlo (MCMC) methodology was performed with 2,000 iterations (Hamilton Monte Carlo; 1,000 warm up, 1,000 sampling the chains). The models were validated running 3 chains (no major differences were found between these), using the Gelman and Rubin diagnostic (R̂ between 1 and 1.02), inspecting the effective number of independent samples form the posterior (worst cases, 100 samples), and comparing the predictions with the original data (see an example in Fig. S1 in the supplemental material). The analysis was performed using R (61) and Stan (62) by using functions provided in reference 63. The data were well described in all models (Fig. S1), and we could determine posterior probability distributions for all four response variables (P. larvae spore levels, AFB symptoms, colony size and brood size [Fig. 1]) and for all calculated differences.

## Supplementary Material

Supplemental file 1

## APPENDIX

### MODEL STRUCTURE OF ALL EIGHT MODELS

Response variable_i_ ∼ Poisson(λ_i_)

Log(λ_i_) = α_Control_ + β_Antibiotic_ Antibiotic_i_ + β_Placebo_ Placebo_i_ + β_hbs-LAB_ hbs-LAB_i_ + α_Colony[i]_ + α_Day[i]_ + α_Observation[i]_

α_Control_ ∼ Normal(0, XX)

β_Antibiotic_ ∼ Normal(0, XX)

β_Placebo_ ∼ Normal(0, XX)

β_hbs-LAB_ ∼ Normal(0, XX)

α_Colony_ ∼ Normal(0, σ_Colony_)

σ_Colony_ ∼ Exp(1)

α_Day_ ∼ Normal(0, σ_Day_)

σ_Day_ ∼ Exp(1)

α_Observation_ ∼ Normal(0, σ_Observation_)

σ_ObservationID_ ∼ Exp(1)

For the models with all six or with only occasions 3 and 4, the XX sigma was as follows: spore count = 10, clinical symptoms = 1, colony size = 1, and brood size = 10.

## References

[1] Cox-Foster DL, Conlan S, Holmes EC, Palacios G, Evans JD, Moran NA, Quan P-L, Briese T, Hornig M, Geiser DM, Martinson V, VanEngelsdorp D, Kalkstein AL, Drysdale A, Hui J, Zhai J, Cui L, Hutchison SK, Simons JF, Egholm M, Pettis JS, Lipkin WI 2007 A metagenomic survey of microbes in honey bee colony collapse disorder. Science 318:283–287. doi:10.1126/science.1146498. 17823314

[2] Corby-Harris V, Maes P, Anderson KE 2014 The bacterial communities associated with honey bee (Apis mellifera) foragers. PLoS One 9:e95056. doi:10.1371/journal.pone.0095056. 24740297 PMC3989306

[3] Cornman RS, Dainat J, De Miranda JR, Doublet V, Emery O, Evans JD, Farinelli L, Schmid-Hempel P, Schmid-Hempel R, Song J, Schwarz RS, Engel P, Kwong WK, McFrederick Q, Anderson KE, Barribeau SM, Chandler JA, Cornman RS, Dainat J, De Miranda JR, Doublet V, Emery O, Evans JD, Farinelli L, Flenniken ML, Granberg F, Grasis JA, Gauthier L, Hayer J, Koch H, Kocher S, Martinson VG, Moran N, Munoz-Torres M, Newton I, Paxton RJ, Powell E, Sadd BM, Schmid-Hempel P, Schmid-Hempel R, Song SJ, Schwarz RS, van Engelsdorp D, Dainat B 2016 The bee microbiome: impact on bee health and model for evolution and ecology of host-microbe interactions. mBio 7:e02164-15. doi:10.1128/mBio.02164-15. 27118586 PMC4850275

[4] Genersch E 2010 American foulbrood in honeybees and its causative agent, Paenibacillus larvae. J Invertebr Pathol 103:S10–S19. doi:10.1016/j.jip.2009.06.015. 19909971

[5] Mill AC, Rushton SP, Shirley MDF, Smith GC, Mason P, Brown MA, Budge GE 2014 Clustering, persistence and control of a pollinator brood disease: epidemiology of American foulbrood. Environ Microbiol 16:3753–3763. doi:10.1111/1462-2920.12292. 24119163

[6] Ebeling J, Knispel H, Hertlein G, Fünfhaus A, Genersch E 2016 Biology of Paenibacillus larvae, a deadly pathogen of honey bee larvae. Appl Microbiol Biotechnol 100:7387–7395. doi:10.1007/s00253-016-7716-0. 27394713

[7] Forsgren E, Locke B, Sircoulomb F, Schäfer MO 2018 Bacterial diseases in honeybees. Curr Clin Microbiol Rep 5:18–25. doi:10.1007/s40588-018-0083-0.

[8] Bailey L, Ball B 1991 Honey bee pathology. Acadamic Press, Cambridge, MA.

[9] Fries I, Camazine S 2001 Implications of horizontal and vertical pathogen transmission for honey bee epidemiology. Apidologie 32:199–214. doi:10.1051/apido:2001122.

[10] Lindström A, Korpela S, Fries I 2008 The distribution of Paenibacillus larvae spores in adult bees and honey and larval mortality, following the addition of American foulbrood diseased brood or spore-contaminated honey in honey bee (Apis mellifera) colonies. J Invertebr Pathol 99:82–86. doi:10.1016/j.jip.2008.06.010. 18640122

[11] Lindström A, Korpela S, Fries I 2008 Horizontal transmission of Paenibacillus larvae spores between honey bee (Apis mellifera) colonies through robbing. Apidologie 39:515–522. doi:10.1051/apido:2008032.

[12] Elzen PJ, Westervelt D, Causey D, Ellis J, Hepburn HR, Neumann P 2002 Method of application of tylosin, an antibiotic for American foulbrood control, with effects on small hive beetle (Coleoptera: Nitidulidae) populations. J Econ Entomol 95:1119–1122. doi:10.1603/0022-0493-95.6.1119. 12539820

[13] Evans JD 2003 Diverse origins of tetracycline resistance in the honey bee bacterial pathogen Paenibacillus larvae. J Invertebr Pathol 83:46–50. doi:10.1016/S0022-2011(03)00039-9. 12725811

[14] Katznelson H 1950 The influence of antibiotics and sulfa drugs on Bacillus larvae, cause of American foulbrood of the honeybee, in vitro and in vivo. J Bacteriol 59:471–479. 15436420 10.1128/jb.59.4.471-479.1950 PMC385787

[15] Oldroyd BP, Goodman RD, Hornitzky MAZ, Chandler D 1989 The effect on American foulbrood of standard oxytetracycline hydrochloride treatments for the control of European foulbrood of honeybees (Apis mellifera). Aust J Agric Res 40:691–697. doi:10.1071/AR9890691.

[16] Pettis JS, Feldlaufer MF 2005 Efficacy of lincomycin and tylosin in controlling American foulbrood in honey bee colonies. J Apic Res 44:106–108. doi:10.1080/00218839.2005.11101158.

[17] Reybroeck W, Daeseleire E, De Brabander HF, Herman L 2012 Antimicrobials in beekeeping. Vet Microbiol 158:1–11. doi:10.1016/j.vetmic.2012.01.012. 22342494

[18] Pătruică S, Mot D, Pǎtruicǎ S, Mot D 2012 The effect of using prebiotic and probiotic products on intestinal micro-flora of the honeybee (Apis mellifera carpatica.). Bull Entomol Res 102:1–5. 22459313 10.1017/S0007485312000144

[19] Sabaté DC, Cruz MS, Benítez-Ahrendts MR, Audisio MC 2012 Beneficial effects of Bacillus subtilis subsp. subtilis Mori2, a honey-associated strain, on honeybee colony performance. Probiotics Antimicrob Proteins 4:39–46. doi:10.1007/s12602-011-9089-0. 26781735

[20] Pǎtruicǎ S, Huţu I 2013 Economic benefits of using prebiotic and probiotic products as supplements in stimulation feeds administered to bee colonies. Turkish J Vet Anim Sci 37:259–263.

[21] Janashia I, Alaux CC 2016 Specific immune stimulation by endogenous bacteria in honey bees (Hymenoptera: Apidae). J Econ Entomol 109:1474–1477. doi:10.1093/jee/tow065. 27063842

[22] Evans JD, Lopez DL 2004 Bacterial probiotics induce an immune response in the honey bee (Hymenoptera: Apidae). J Econ Entomol 97:752–756. doi:10.1093/jee/97.3.752. 15279248

[23] Maggi M, Negri P, Plischuk S, Szawarski N, De Piano F, De Feudis L, Eguaras M, Audisio C 2013 Effects of the organic acids produced by a lactic acid bacterium in Apis mellifera colony development, Nosema ceranae control and fumagillin efficiency. Vet Microbiol 167:474–483. doi:10.1016/j.vetmic.2013.07.030. 23978352

[24] Killer J, Dubná S, Sedláček I, Švec P 2014 Lactobacillus apis sp. nov., from the stomach of honeybees (Apis mellifera), having an in vitro inhibitory effect on the causative agents of American and European foulbrood. Int J Syst Evol Microbiol 64:152–157. doi:10.1099/ijs.0.053033-0. 24096349

[25] Forsgren E, Olofsson TC, Vásquez A, Fries I 2010 Novel lactic acid bacteria inhibiting Paenibacillus larvae in honey bee larvae. Apidologie 41:99–108. doi:10.1051/apido/2009065.

[26] Olofsson TC, Vásquez A 2008 Detection and identification of a novel lactic acid bacterial flora within the honey stomach of the honeybee Apis mellifera. Curr Microbiol 57:356–363. doi:10.1007/s00284-008-9202-0. 18663527

[27] Olofsson TC, Alsterfjord M, Nilson B, Butler E, Vasquez A 2014 Lactobacillus apinorum sp. nov., Lactobacillus mellifer sp. nov., Lactobacillus mellis sp. nov., Lactobacillus melliventris sp. nov., Lactobacillus kimbladii sp. nov., Lactobacillus helsingborgensis sp. nov. and Lactobacillus kullabergensis sp. nov., isol. Int J Syst Evol Microbiol 64:3109–3119. doi:10.1099/ijs.0.059600-0. 24944337 PMC4156108

[28] Makarova KS, Koonin EV 2007 Evolutionary genomics of lactic acid bacteria. J Bacteriol 189:1199–1208. doi:10.1128/JB.01351-06. 17085562 PMC1797341

[29] Butler È, Alsterfjord M, Olofsson TC, Karlsson C, Malmström J, Vásquez A 2013 Proteins of novel lactic acid bacteria from Apis mellifera mellifera: an insight into the production of known extra-cellular proteins during microbial stress. BMC Microbiol 13:235. doi:10.1186/1471-2180-13-235. 24148670 PMC4015849

[30] Lamei S, Stephan JG, Riesbeck K, Vasquez A, Olofsson T, Nilson B, de Miranda JR, Forsgren E 2019 The secretome of honeybee-specific lactic acid bacteria inhibits Paenibacillus larvae growth. J Apic Res 58:405–412. doi:10.1080/00218839.2019.1572096.

[31] Evans JD, Aronstein K, Chen YP, Hetru C, Imler J-L, Jiang H, Kanost M, Thompson GJ, Zou Z, Hultmark D 2006 Immune pathways and defence mechanisms in honey bees Apis mellifera. Insect Mol Biol 15:645–656. doi:10.1111/j.1365-2583.2006.00682.x. 17069638 PMC1847501

[32] Rauch S, Ashiralieva A, Hedtke K, Genersch E 2009 Negative correlation between individual-insect-level virulence and colony-level virulence of Paenibacillus larvae, the etiological agent of american foulbrood of honeybees. Appl Environ Microbiol 75:3344–3347. doi:10.1128/AEM.02839-08. 19304833 PMC2681656

[33] Evans JD, Spivak M 2010 Socialized medicine: individual and communal disease barriers in honey bees. J Invertebr Pathol 103:S62–S72. doi:10.1016/j.jip.2009.06.019. 19909975

[34] Alberoni D, Gaggìa F, Baffoni L, Di Gioia D 2016 Beneficial microorganisms for honey bees: problems and progresses. Appl Microbiol Biotechnol 100:9469–9482. doi:10.1007/s00253-016-7870-4. 27717968

[35] Ptaszyńska AA, Borsuk G, Zdybicka-Barabas A, Cytryńska M, Małek W 2016 Are commercial probiotics and prebiotics effective in the treatment and prevention of honeybee nosemosis C? Parasitol Res 115:397–406. doi:10.1007/s00436-015-4761-z. 26437644 PMC4700093

[36] Forsgren E, Fries I 2010 Comparative virulence of Nosema ceranae and Nosema apis in individual European honey bees. Vet Parasitol 170:212–217. doi:10.1016/j.vetpar.2010.02.010. 20299152

[37] Kawasaki S, Kurosawa K, Miyazaki M, Yagi C, Kitajima Y, Tanaka S, Irisawa T, Okada S, Sakamoto M, Ohkuma M, Niimura Y 2011 Lactobacillus floricola sp. nov., lactic acid bacteria isolated from mountain flowers. Int J Syst Evol Microbiol 61:1356–1359. doi:10.1099/ijs.0.022988-0. 20601482

[38] Louie A, VanScoy BD, Brown DL, Kulawy RW, Heine HS, Drusano GL 2012 Impact of spores on the comparative efficacies of five antibiotics for treatment of Bacillus anthracis in an in vitro hollow fiber pharmacodynamic model. Antimicrob Agents Chemother 56:1229–1239. doi:10.1128/AAC.01109-10. 22155821 PMC3294912

[39] Murray KD, Aronstein KA 2006 Oxytetracycline-resistance in the honey bee pathogen Paenibacillus larvae is encoded on novel plasmid pMA67. J Apic Res 45:207–214. doi:10.1080/00218839.2006.11101349.

[40] Alippi AM, López AC, Reynaldi FJ, Grasso DH, Aguilar OM 2007 Evidence for plasmid-mediated tetracycline resistance in Paenibacillus larvae, the causal agent of American foulbrood (AFB) disease in honeybees. Vet Microbiol 125:290–303. doi:10.1016/j.vetmic.2007.05.018. 17601687

[41] Li JH, Evans JD, Li WF, Zhao YZ, DeGrandi-Hoffman G, Huang SK, Li ZG, Hamilton M, Chen YP 2017 New evidence showing that the destruction of gut bacteria by antibiotic treatment could increase the honey bee’s vulnerability to Nosema infection. PLoS One 12:e0187505. doi:10.1371/journal.pone.0187505. 29125851 PMC5681286

[42] Cantwell G 1980 The use of ethylene oxide to fumigate honey bee equipment in the United states and Canada during the 1970s. Am Bee J 120:840–843.

[43] Bolger P, Pharo H 2014 Control of American foulbrood in New Zealand, p 167–171. *In* Ritter W (ed), Bee health and veterinarians. World Organisation for Animal Health, Paris, France.

[44] Huang W-F, Solter LF, Yau PM, Imai BS 2013 Nosema ceranae escapes fumagillin control in honey bees. PLoS Pathog 9:e1003185. doi:10.1371/journal.ppat.1003185. 23505365 PMC3591333

[45] Mendoza Y, Diaz-Cetti S, Ramallo G, Santos E, Porrini M, Invernizzi C 2017 Nosema ceranae winter control: study of the effectiveness of different fumagillin treatments and consequences on the strength of honey bee (Hymenoptera: Apidae) colonies. J Econ Entomol 110:1–5. doi:10.1093/jee/tow228. 28025388

[46] Liu F, He J, Fu W 2005 Highly controlled nest homeostasis of honey bees helps deactivate phenolics in nectar. Naturwissenschaften 92:297–299. doi:10.1007/s00114-005-0629-x. 15856150

[47] Wu-Smart J, Spivak M 2016 Sub-lethal effects of dietary neonicotinoid insecticide exposure on honey bee queen fecundity and colony development. Sci Rep 6:32108. doi:10.1038/srep32108. 27562025 PMC4999797

[48] Raymann K, Bobay L-M, Moran NA 2018 Antibiotics reduce genetic diversity of core species in the honeybee gut microbiome. Mol Ecol 27:2057–2066. doi:10.1111/mec.14434. 29164717 PMC5935549

[49] Raymann K, Shaffer Z, Moran NA 2017 Antibiotic exposure perturbs the gut microbiota and elevates mortality in honeybees. PLoS Biol 15:e2001861. doi:10.1371/journal.pbio.2001861. 28291793 PMC5349420

[50] Zheng H, Powell JE, Steele MI, Dietrich C, Moran NA 2017 Honeybee gut microbiota promotes host weight gain via bacterial metabolism and hormonal signaling. Proc Natl Acad Sci U S A 114:4775–4780. doi:10.1073/pnas.1701819114. 28420790 PMC5422775

[51] Janashia I, Choiset Y, Jozefiak D, Déniel F, Coton E, Moosavi-Movahedi AA, Chanishvili N, Haertlé T 2018 Beneficial protective role of endogenous lactic acid bacteria against mycotic contamination of honeybee beebread. Probiotics Antimicrob Proteins 10:638–646. doi:10.1007/s12602-017-9379-2. 29297160

[52] Vásquez A, Forsgren E, Fries I, Paxton RJ, Flaberg E, Szekely L, Olofsson TC 2012 Symbionts as major modulators of insect health: lactic acid bacteria and honeybees. PLoS One 7:e33188. doi:10.1371/journal.pone.0033188. 22427985 PMC3299755

[53] Butler E, Oien RF, Lindholm C, Olofsson TC, Nilson B, Vásquez A, Butler È, Oien RF, Lindholm C, Olofsson TC, Nilson B, Vásquez A 2016 A pilot study investigating lactic acid bacterial symbionts from the honeybee in inhibiting human chronic wound pathogens. Int Wound J 13:729–737. doi:10.1111/iwj.12360. 25196349 PMC7950084

[54] Pettis JS, Rose R, Chaimanee V 2017 Chemical and cultural control of Tropilaelaps mercedesae mites in honeybee (Apis mellifera) colonies in Northern Thailand. PLoS One 12:e0188063. doi:10.1371/journal.pone.0188063. 29125881 PMC5681254

[55] Delaplane KS, van der Steen J, Guzman-Novoa E 2013 Standard methods for estimating strength parameters of Apis mellifera colonies. J Apic Res 52:1–12. doi:10.3896/IBRA.1.52.1.03.

[56] de Graaf DC, Alippi AM, Antúnez K, Aronstein KA, Budge G, De Koker D, De Smet L, Dingman DW, Evans JD, Foster LJ, Fünfhaus A, Garcia-Gonzalez E, Gregore A, Human H, Murray KD, Nguyen BK, Poppinga L, Spivak M, van Engelsdorp D, Wilkins S, Genersch E 2013 Standard methods for American foulbrood research. J Apic Res 52:1–28. doi:10.3896/IBRA.1.52.1.11. PMC3816652 24198438

[57] Forsgren E, Laugen AT 2014 Prognostic value of using bee and hive debris samples for the detection of American foulbrood disease in honey bee colonies. Apidologie 45:10–20. doi:10.1007/s13592-013-0225-6.

[58] McElreath R 2015 Statistical rethinking: a Bayesian course with examples in R and Stan. Chapman & Hall/CRC, Boca Raton, FL.

[59] Kruschke JK, Liddell TM 2016 The Bayesian new statistics: hypothesis testing, estimation, meta-analysis, and planning from a Bayesian perspective. Psychon Bull Rev 25:1–53. doi:10.3758/s13423-016-1221-4. 28176294

[60] Harrison XA 2014 Using observation-level random effects to model overdispersion in count data in ecology and evolution. PeerJ 2:e616. doi:10.7717/peerj.616. 25320683 PMC4194460

[61] R Core Team 2017 R: a language and environment for statistical computing. R Foundation for Statistical Computing, Vienna, Austria.

[62] Stan Development Team. 2017 Stan modeling language users guide and reference manual, version 2.17.0. http://mc-stan.org.

[63] McElreath R 2016 Rethinking: an R package for fitting and manipulating Bayesian models. http://xcelab.net/R/rethinking_package.pdf.

[64] Hasemann L 1961 How long can spores of American foulbrood live? Am Bee J 101:298–299.

[65] Locke B, Low M, Forsgren E 2019 An integrated management strategy to prevent outbreaks and eliminate infection pressure of American foulbrood disease in a commercial beekeeping operation. Prev Vet Med 167:48–52. doi:10.1016/j.prevetmed.2019.03.023. 31027721

[66] De Piano FG, Maggi M, Pellegrini MC, Cugnata NM, Szawarski M, Buffa F, Negri P, Fuselli SR, Audisio CM, Ruffinengo SR 2017 Effects of Lactobacillus johnsonii AJ5 metabolites on nutrition, nosema ceranae development and performance of Apis mellifera L. J Apic Sci 61:93–104. doi:10.1515/JAS-2017-0007.

