# Supplementary material for: Honeybee-Specific Lactic Acid Bacterium Supplements Have No Effect on American Foulbrood-Infected Honeybee Colonies
Source: Appl Environ Microbiol. 2019 Jun 17;85(13):e00606-19. doi: 10.1128/AEM.00606-19 (PMC6581185; doi:10.1128/AEM.00606-19)
Supplement: Supplemental file 1 [file AEM.00606-19-s0001.pdf]

Supplement

Title:

Honeybee-specific lactic acid bacterial supplements have no effect on American foulbrood infected honeybee colonies

Running title: Effect of lactic acid bacteria and tylosin on AFB

Jörg G Stephan[a,#,$], Sepideh Lamei[a,b,d,$], Jeffery S. Pettis[c,e], Kristian Riesbeck[b], Joachim R. de Miranda[a], Eva Forsgren[a]

[a] Department of Ecology, Swedish University of Agricultural Sciences, Uppsala, 750 07 Sweden

[b] Clinical Microbiology, Department of Translational Medicine, Faculty of Medicine, Lund University, Malmö, 205 02 Sweden

[c] USDA ARS, Building 306, Beltsville Agricultural Research Center-East, Beltsville, MD, 20705, USA

[d] present address: Recipharm AB, Karlskoga, Sweden

[e] present address: Pettis and Assoc. LLC, Salisbury MD, USA

# Corresponding author

$ Contributed equally to the manuscript

Model structure of all eight models

$\text{Response variable}_i \sim \text{Poisson}(\lambda_i)$

$\text{Log}(\lambda_i) = \alpha_{Control} + \beta_{Antibiotic}\ \text{Antibiotic}_i + \beta_{Placebo}\ \text{Placebo}_i + \beta_{hbs\text{-}LAB}\ \text{hbs-LAB}_i +$

$\alpha_{Colony[i]} + \alpha_{Day[i]} + \alpha_{Observation[i]}$

$\alpha_{Control} \sim \text{Normal}(0,XX)$

$\beta_{Antibiotic} \sim \text{Normal}(0,XX)$

$\beta_{Placebo} \sim \text{Normal}(0,XX)$

$\beta_{hbs\text{-}LAB} \sim \text{Normal}(0,XX)$

$\alpha_{Colony} \sim \text{Normal}(0, \sigma_{Colony})$

$\sigma_{Colony} \sim \text{Exp}(1)$

$\alpha_{Day} \sim \text{Normal}(0, \sigma_{Day})$

$\sigma_{Day} \sim \text{Exp}(1)$

$\alpha_{Observation} \sim \text{Normal}(0, \sigma_{Observation})$

$\sigma_{ObservationID} \sim \text{Exp}(1)$

XX: For the models with all six or only with occasion 3 and 4 this sigma was as follows:

Spore count = 10; Clinical symptoms = 1; Colony size = 1; Brood size = 10

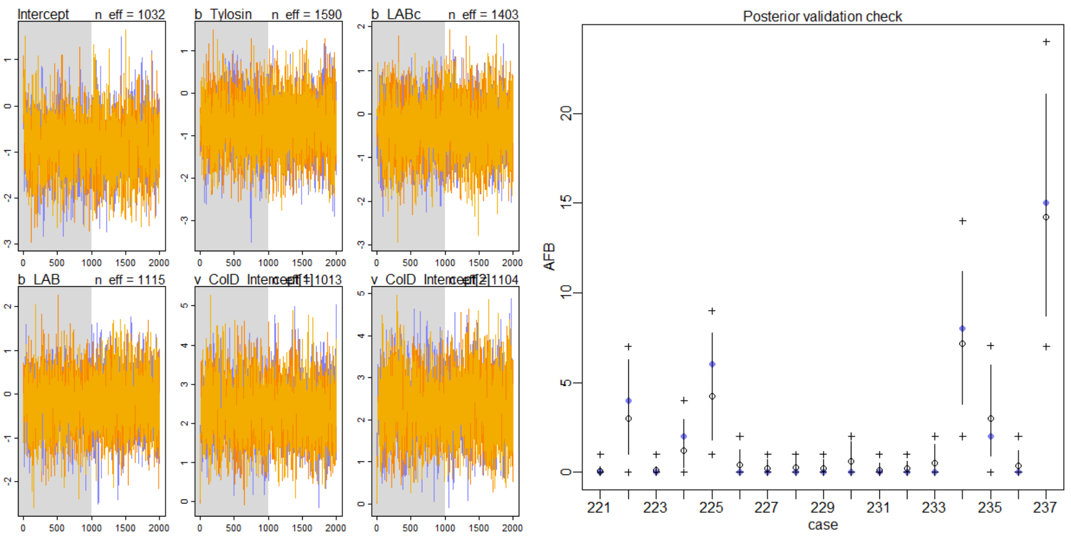

Fig. S1: Examples of model diagnostic plots. On the left side chains from 6 parameters (for the intercept, 4 treatments, and 2 individual colonies) from the model on clinical symptoms at all sampling occasions are shown (warm up in grey, sampling in white, 3 chains in different colors). On the right some of the posterior for the clinical symptoms for all sampling occasions are shown with: blue points = original data, circles = posterior mean with 89% percentile intervals, + symbols = 89% intervals of predicted scores.

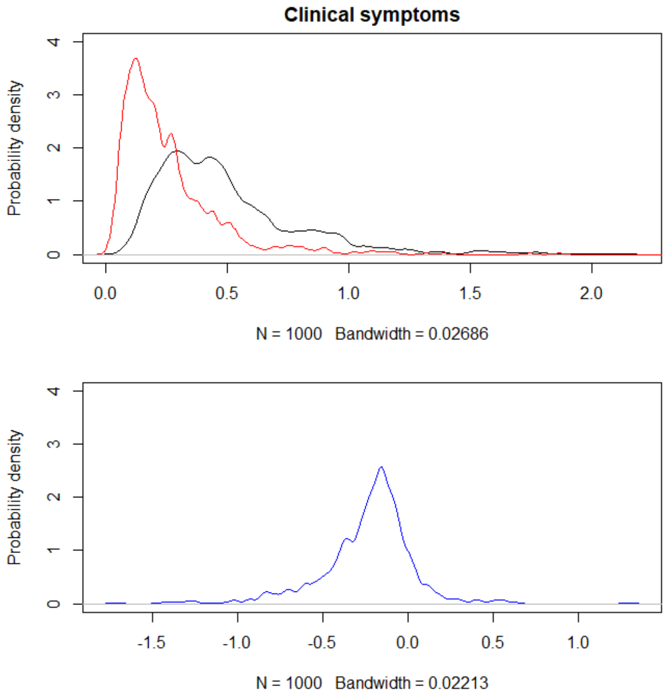

Fig. S2: Examples of posterior and calculation of differences. On top the allocation of credibility across probable values for the parameter given the data are shown for tylosin (red) and the control (black). These distributions are summarized with mean and crediebility inervals (tables and Fig. 2: Estimate). We used the 89% highest posterior density intervals which contains the parameter values of highest probability to describe the distribution. The probability distribution for the difference between both can be calculated by subtracting one distribution from the other (see: Difference in Fig. 2). This is shown in the bottom and also represents how likely each value is. In all comparisons we subtracted the control from the treatment. In this example most of the values (89% of the area) are below zero indicating a decrease from tylosin to control hence giving a proability of 89% that the difference is small than zero.
